# Differential Immune Response to Bioprosthetic Heart Valve Tissues in the α1,3Galactosyltransferase-Knockout Mouse Model

**DOI:** 10.3390/bioengineering10070833

**Published:** 2023-07-13

**Authors:** Kelly Casós, Roger Llatjós, Arnau Blasco-Lucas, Sebastián G. Kuguel, Fabrizio Sbraga, Cesare Galli, Vered Padler-Karavani, Thierry Le Tourneau, Marta Vadori, Andrea Perota, Jean-Christian Roussel, Tomaso Bottio, Emanuele Cozzi, Jean-Paul Soulillou, Manuel Galiñanes, Rafael Máñez, Cristina Costa

**Affiliations:** 1 Infectious Diseases and Transplantation Division, Institut d’Investigació Biomèdica de Bellvitge [IDIBELL], L’Hospitalet de Llobregat, 08908 Barcelona, Spain; 2 Pathology Department, Bellvitge University Hospital, L’Hospitalet de Llobregat, 08907 Barcelona, Spain; 3 Cardiac Surgery Department, Bellvitge University Hospital, L’Hospitalet de Llobregat, 08907 Barcelona, Spain; 4 Avantea Srl, 26100 Cremona, Italy; 5 Department of Cell Research and Immunology, The Shmunis School of Biomedicine and Cancer Research, The George S. Wise Faculty of Life Sciences, Tel Aviv University, Tel Aviv 6997801, Israel; 6 Institut du Thorax, INSERM UMR1087, Nantes University Hospital, 44093 Nantes, France; 7 Transplantation Immunology Unit, Padua University Hospital, 35128 Padova, Italy; 8 Department of Cardiac, Thoracic, Vascular Sciences and Public Health, University of Padua Medical School, 35121 Padova, Italy; 9 Institut de Transplantation-Urologie-Néphrologie, INSERM Unité Mixte de Recherche 1064, Nantes University Hospital, 44093 Nantes, France; 10 Department of Cardiac Surgery and Reparative Therapy of the Heart, Vall d’Hebron Research Institute [VHIR], University Hospital Vall Hebron, Universitat Autònoma de Barcelona, 08035 Barcelona, Spain; 11 Intensive Care Department, Bellvitge University Hospital, L’Hospitalet de Llobregat, 08907 Barcelona, Spain

**Keywords:** bioprosthetic heart valves, structural valve deterioration, α1,3-galactosyltransferase-knockout mice, anti-Gal antibodies, cellular immune infiltrate

## Abstract

Structural valve deterioration (SVD) of bioprosthetic heart valves (BHVs) has great clinical and economic consequences. Notably, immunity against BHVs plays a major role in SVD, especially when implanted in young and middle-aged patients. However, the complex pathogenesis of SVD remains to be fully characterized, and analyses of commercial BHVs in standardized-preclinical settings are needed for further advancement. Here, we studied the immune response to commercial BHV tissue of bovine, porcine, and equine origin after subcutaneous implantation into adult α1,3-galactosyltransferase-knockout (Gal KO) mice. The levels of serum anti-galactose α1,3-galactose (Gal) and -non-Gal IgM and IgG antibodies were determined up to 2 months post-implantation. Based on histological analyses, all BHV tissues studied triggered distinct infiltrating cellular immune responses that related to tissue degeneration. Increased anti-Gal antibody levels were found in serum after ATS 3f and Freedom/Solo implantation but not for Crown or Hancock II grafts. Overall, there were no correlations between cellular-immunity scores and post-implantation antibodies, suggesting these are independent factors differentially affecting the outcome of distinct commercial BHVs. These findings provide further insights into the understanding of SVD immunopathogenesis and highlight the need to evaluate immune responses as a confounding factor.

## 1. Introduction

The implantation of bioprosthetic heart valves (BHVs) has increased because of population aging and greater longevity [1,2]. Research in the area is also very active because of their potential application in young people [as those affected by rheumatic valve disease] [3]. Moreover, the advantages provided by the high biological and hemodynamic compatibility of BHV tissues relative to mechanical valves, together with less thrombogenicity, further motivate research and development for its use in surgical heart-valve replacement [4,5], transcatheter aortic valve implantation (TAVI) [6,7] and other devices [8,9]. However, structural valve deterioration (SVD) limits their application and long-term function [1,3,4,5,6,10]. The pathogenesis of SVD is not fully elucidated, but it is known to be multifactorial [4,11,12]. Recently, the role of the immune response has gained much attention as a mediator for SVD and a potential target for intervention [4,12,13,14,15].

The most common tissues used for BHVs are of animal origin, basically from bovine and porcine pericardium, pig valves, and occasionally from equine pericardium [4,15,16]. Many are commercialized by large companies that produce BHVs following not-fully disclosed procedures that modify their structure and function. Glutaraldehyde fixation is a basic step for the preparation of the BHV that provides tissue stability and reduces immunogenicity [4,17]. However, it has been associated with BHV calcification and SVD [4,17]. To overcome this problem, anti-calcification treatments are applied after fixation to the new-generation BHVs. Despite these efforts and improvements, residual immunogenic carbohydrates have been detected in commercial BHVs [18,19,20]. In particular, the galactose α1,3-galactose (Gal) and N-glycolylneuraminic acid (Neu5Gc) antigens are present at various levels in commercial BHVs made of porcine valves, bovine or equine pericardium [18,19,20]. Humans do not produce Gal and Neu5Gc antigens and, in turn, generate natural antibodies that recognize them. Furthermore, human antibody reactivity towards BHVs has been detected after incubation with human sera [18,20,21], as well as increases in anti-Gal and anti-Neu5Gc antibodies in patients receiving BHVs after heart valve replacement [14,21,22,23,24]. Notably, genetic modification of pigs and cattle is already available with the potential to generate BHVs devoid of these carbohydrate antigens [20,25,26,27].

Despite the advances in the generation of better BHVs, the actual immunogenicity of currently used BHVs and the effect on their durability is still not well established [28]. As a result, millions of patient lives depend on the long-term performance of BHVs, but surgeons have limited information to select the most appropriate BHV. In this regard, standardized animal models could be a valuable tool to characterize and compare the responses to BHV tissues, helping to elucidate the pathogenesis of SVD and to test new therapeutic strategies. Although the data generated could not be directly extrapolated to the clinical setting [13], it may also provide additional information for the surgeons’ consideration.

In this work, we assessed the feasibility of a simple subcutaneous model using adult α1,3-galactosyltransferase knockout (Gal KO) mice to study the humoral and cellular immune responses toward different commercial BHVs. The age of mice was selected to stand for the age range of patients that pose a greater difficulty in choosing BHV over mechanical heart valves because of SVD [29].

## 2. Materials and Methods

### 2.1. Tissue Samples and Processing

The commercial BHVs used in this work were kindly donated by Medtronic (ATS 3f, Hancock II) and Sorin-LivaNova (Crown, Freedom Solo, and Pericarbon Freedom) and kept in their original conditions prior to experimental assessment. The human pericardium was obtained from a patient subjected to cardiac surgery and fixed with 0,6% glutaraldehyde (Merck KGaA, Darmstadt, Germany) in phosphate-buffered saline (PBS, Merck) for one month and subsequently maintained at 0.2% glutaraldehyde. Prior to surgical implantation, small pieces of BHV and human pericardium were generated under sterile conditions, their weight recorded, and extensively washed in tissue-culture-grade PBS. The selected pieces were kept overnight in PBS in sterile 24-well plates. In compliance with current legislation, this project was approved by the ethical clinical committee of Bellvitge University Hospital.

### 2.2. Mouse Model of BHV Tissue Implantation

The procedures and the care of mice complied with European Commission guidelines and were approved by the local institutional ethical committee and Generalitat de Catalunya. For these studies, we used mice knockout for α1,3-galactosyltransferase (Gal KO) from our colony (B6xCBAx129sv mixed background) [30], most of which develop natural anti-Gal antibodies with age. A pilot study was first conducted with adult Gal KO mice implanted subcutaneously with ATS 3f or Pericarbon Freedom for 2 (*n* = 5, 12–14 months-old mice) and 4 months (*n* = 6, 10–12 months-old mice). One mouse (M3) was lost for causes unrelated to graft rejection (during blood collection). Next, the experimental setting was established with adult mice (10–14 months old) for a 2-month implantation period. Mice were equally distributed between 5 experimental groups (*n* = 7–10), including both males and females, as well as mice with different titers of anti-Gal IgM and IgG antibodies pre-determined by ELISA using plates coated with Gal-human serum albumin (HSA) as previously described [30]. These 5 mouse cohorts were set to study the immune response against four commercial BHVs: ATS 3f, Crown [same tissue as Mitroflow PRT), Freedom Solo, or Pericarbon Freedom (pooled and named Freedom here because both prostheses are made with the same tissue type and methodological preparation), and Hancock II, and human pericardium fixed in 0.6% glutaraldehyde for comparison (Table 1). All the tissues, either the pericardium or the cusps of Hancock II, were cut into small pieces controlled by wet weight (7–10 mg). A single piece of tissue was then implanted subcutaneously on the dorsum of each mouse under isofluorane anesthesia and sterile conditions. Blood was collected, and body weight was recorded one week before implantation and then at 2 weeks and 2 months after surgery. Unless indicated, the experiments concluded at two months, and the tissue implant was retrieved. Blood was allowed to clot for 1 h, and sera were obtained and stored for later determination of xenoantibody levels.

### 2.3. Pathology

The grafts were examined macroscopically at the time of collection, fixed in 10% neutral-buffered formalin (Merck, Darmstadt, Germany), processed, and embedded in paraffin for histologic examination. Sections of 3 µm were cut, stained with hematoxylin and eosin (H&E) following standard procedures, and evaluated independently and blindly by two investigators, including an expert pathologist, for signs of immune rejection. A scoring system [31] of 1–5 was established to provide a semi-quantification of the amount of cellular immune infiltrate (detailed in figure legend). To assess the proportion of BHV tissue affected and preserved, pictures taken at 100× of the stained sections corresponding to the pilot study were analyzed by ImageJ software (U. S. National Institutes of Health, Bethesda, MD, USA). Briefly, the areas of the full BHV tissue and that with the preserved structure were delimited, measured, and the percentage calculated.

### 2.4. Culture of Endothelial Cells

Porcine aortic endothelial cells (PAEC) were obtained from the European Collection of Cell Cultures (Porton Down, UK) and cultured in cell-culture conditions in DMEM/10% FBS supplemented with 100 IU/mL penicillin–100 μg/mL streptomycin and 50 μg/mL endothelial cell growth supplement (Millipore, Merck). Culture flasks (TPP Techno Plastic Products AG, Trasadingen, Switzerland) pre-coated with 1% porcine collagen (Merck) in PBS were used.

### 2.5. Determinations of Serum Xenoantibodies

Anti-Gal IgM and IgG antibodies were determined by ELISA, as previously described [30]. Furthermore, we also developed an in-house method for determining xenoantibody reactivity, anti-Gal and -non-Gal antibodies, by flow cytometry. Briefly, confluent PAEC were harvested with TripLE Express (Thermo Fisher Scientific, Waltham, MA, USA), washed, and used to detect possible carbohydrate reactivities. To this end, mouse sera diluted at 1% or 0.5% in PBS 1% bovine serum albumin (Merck) were incubated with PAEC alone or with saturating concentrations of GAS914 (0.5 mg/mL, Novartis, Basel, Switzerland) for 30 min at 4 °C. GAS914 binds anti-Gal antibodies and blocks their reactivity [32]. IgM and IgG reactivities were measured using the secondary antibodies goat anti-mouse IgM Alexa fluor 647 (1/200 dilution) and goat anti-mouse IgG PE (1/150 dilution) (both from Thermo Fisher Scientific) and a Gallios flow cytometer with Kaluza software (Beckman Coulter, Brea, CA, USA). The mean fluorescence intensity (MFI) was determined (for each individual and time point) after subtracting the background established with the secondary antibody alone or with sera with GAS914. The reactivity remaining following GAS914 competition was considered to be associated with anti-non-Gal antibodies.

### 2.6. Statistical Analyses

Statistical analyses were conducted after confirming data normality with the Shapiro–Wilk normality test. Then, the following tools were used as necessary: the one-way ANOVA and Tukey’s test for multiple comparisons, one-way ANOVA and Dunnett’s tests to compare multiple conditions relative to baseline, and paired Student *t* test for single comparisons. The coefficient of determination (R^2^) was calculated for correlations. Statistical analyses were performed using Graph Pad Prism 6. Differences were considered statistically significant at *p* ≤ 0.05.

## 3. Results

### 3.1. Experimental Setting and Immune-Response Kinetics in Gal KO Mice Grafted with BHV Tissue

The Gal KO mice used develop anti-Gal IgM and IgG antibodies spontaneously but with high variability [30]. As a start, we conducted a pilot study with mice of both genders and different levels of serum natural anti-Gal antibody titers as these variables may impact their antibody and overall immune response to the graft. Mice were not subjected to any additional immunization. Body weight (Table 2) and serum antibody titers (Figures S1 and S2, Table S1) were determined up to 4 months post-implantation for two different BHV tissues (ATS 3f made of equine pericardium and Freedom made of bovine pericardium). No major effect on well-being was observed during the period of BHV-tissue implantation for the various cohorts, with the exception of a transitory reduction in body weight after surgery that equally affected both groups (Table 2).

#### 3.1.1. Time Course of the Antibody Response

To assess the antibody response over time, we first determined serum anti-Gal antibody titers up to 2 months post-implantation (pilot study) by ELISA [30]. Two different serum dilutions (1 and 0.5%) were assessed. Results at 0.5% serum were more reliable, with no indication of saturation (Figure S1). Mice with low or no titers of anti-Gal antibodies at baseline maintained the same low anti-Gal reactivity throughout the entire 2 months period, independently of the type of BHV tissue implanted. For mice with higher titers of natural anti-Gal antibodies at baseline, the ELISA detected some mild increases in IgM after implantation. However, no consistent elevations in anti-Gal IgG were detected with this assay (Figure S1). In fact, serum reactivity for both anti-Gal IgM and IgG somehow decreased over time for some mice. To gain more insight, an additional assay was developed to assess the anti-Gal and the overall xenoantibody response. To this end, mouse sera (1 and 0.5%) were incubated with PAEC with and without GAS914 (to block anti-Gal antibodies), and xenoantibody reactivity was measured by flow cytometry. Good staining was attained with this system for both IgM and IgG isotypes (Figure S2), although IgM responses were more pronounced. The serum IgM antibody reactivity from all grafted mice was mostly dependent on anti-Gal antibodies. Indeed, reactivity increased over time after implantation (up to the 9 weeks tested) and was greatly reduced by pre-treatment of sera with GAS914 at all time points in the two cohorts studied. Elevations in anti-Gal and anti-non-Gal IgG reactivity were also observed for some implanted mice (Figure S2). Notably, most overall antibody responses had reached the plateau at 2 months post-implantation (Table S1). A critical part of this work was thus setting up the conditions to assess the anti-Gal antibody levels. Gal KO mice display weaker anti-Gal antibody responses than humans [30,33]. Accordingly, the difficulty in detecting a consistent antibody response against small xenografts processed to reduce their immunogenicity led us to develop a method based on flow cytometry. The underlying rationale was that carbohydrates are exposed in their native form on the cell surface, and flow cytometry allows to detection of antibody reactivity with high sensitivity. To determine the specificity of the antibodies for the Gal antigen, we took advantage of GAS914, which selectively blocks anti-Gal antibodies [32]. After comparing this method with the ELISA routinely performed [30], we chose the flow cytometric measurements for their higher sensitivity. Furthermore, the same assay allowed the measurement of anti-non-Gal antibody reactivity. Finally, based on the data generated in the pilot experiments, we determined that 2 months was long enough to detect the elicited xenoantibody responses in this mouse model.

#### 3.1.2. Time Course of the Histopathological Changes

**Figure 1:**
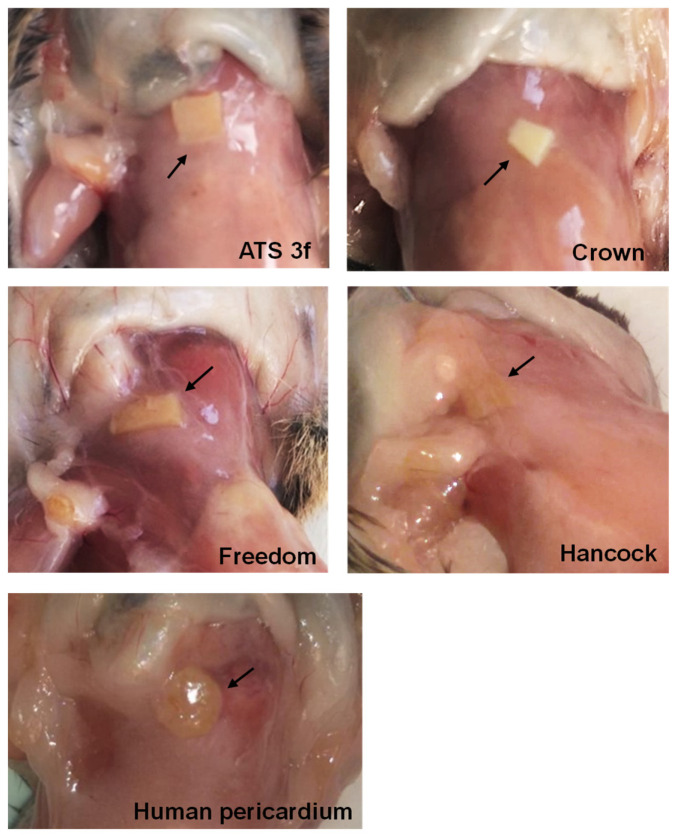
Pathological studies of BHV tissues in adult Gal KO mice at 2 months post-implantation. Representative images of macroscopic examination of adult mice grafted with ATS 3f (*n* = 10), Crown (*n* = 8), Pericarbon Freedom/Freedom Solo (Freedom, *n* = 9), Hancock II (Hancock, *n* = 7), and human pericardium (*n* = 7). The BHV-tissue locations are indicated with arrows.

**Figure 2:**
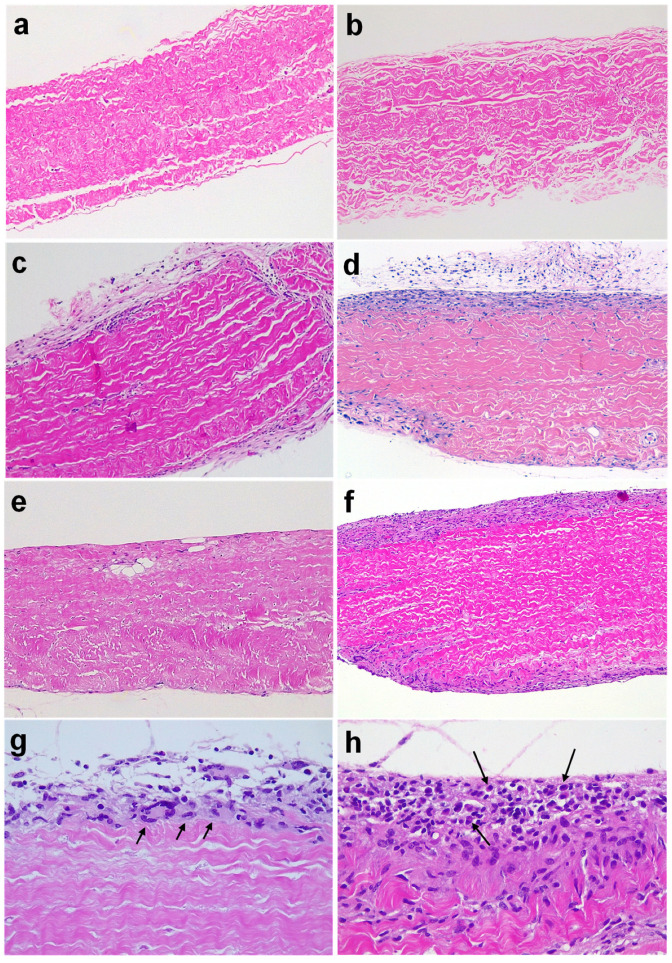
Histological analyses of BHV tissues explanted from adult Gal KO mice up to 4 months post-implantation. The percentage of structurally preserved BHV tissue was calculated for the shown samples (**a**–**f**). Representative images of H&E-stained paraffin sections: non-implanted ATS 3f (**a**) and Freedom Solo (**b**) (100% of both BHV tissues preserved); ATS 3f ((**c**) 86.9% preserved) and Freedom Solo ((**d**) 73.9% preserved) explanted at 2 months post-implantation; ATS 3f ((**e**) 90% preserved) and Freedom Solo ((**f**) 72.2% preserved) explanted at 4 months post-implantation; macrophages and giant cells in 2 months-explanted ATS 3f ((**g**) see arrows); plasma cells in 2-month-explanted Freedom Solo ((**h**) see arrows). Original magnification 100× (**a**–**f**) and 400× (**g**,**h**).

Grafts were surgically retrieved at 2 and 4 months post-implantation. Gross examination at 2 months showed that implants were well preserved and surrounded by the vascularized soft tissue of the recipient (Figure 1). Histological analysis was subsequently conducted after H&E staining of paraffinated tissue (Figure 2). The internal tissue structure was intact with well-organized fibers and the presence of nuclei as in non-implanted tissue (Figure 2a,b,g). However, a cellular immune response was observed with a predominance of macrophages progressing from the edges into the explanted pieces (Figure 2c–h). Some lymphocytes, plasma cells, and a few giant cells (generated by the fusion of macrophages against the tissue) were also found within the immune infiltrate (Figure 2g,h). Overall, the amount of cellular immune infiltrate observed in retrieved BHV tissues was higher at 2 months post-implantation (Figure 2c–f), whereas loss of structure and tissue quality became readily visible in some cases after 4 months. Furthermore, the loss of BHV tissue paralleled the amount of cellular immune infiltrates as was calculated by image analysis of representative pictures (Figure 2a–f).

### 3.2. Gal KO Mice Develop a Cellular Immune Response against BHV Tissues

**Figure 3:**
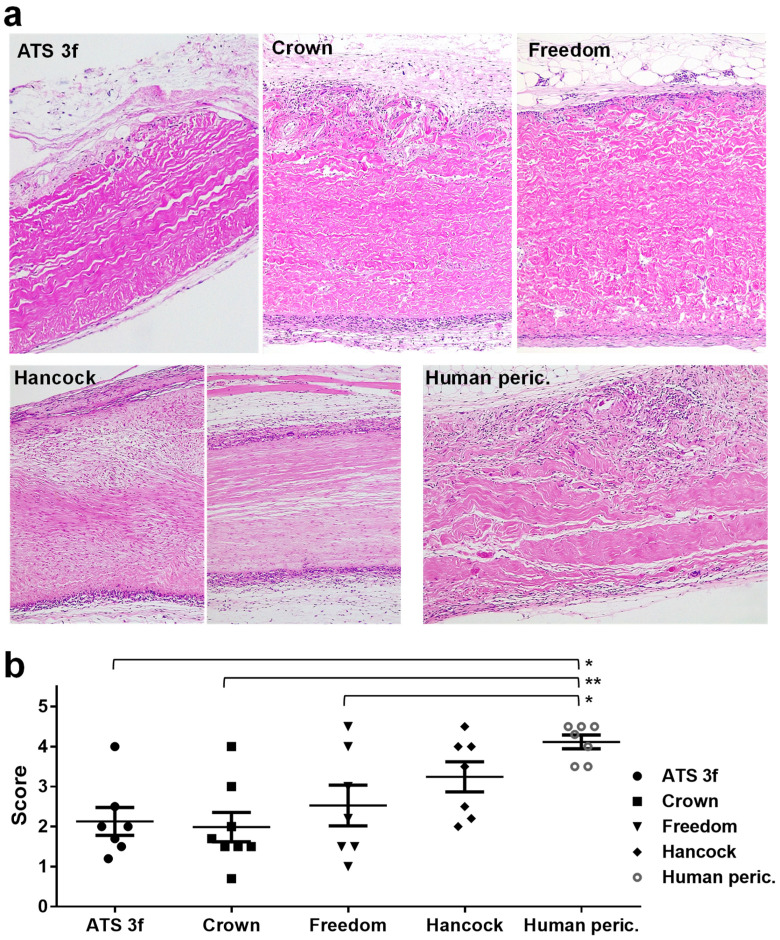
Histological analysis of BHV tissues explanted from adult Gal KO mice at 2 months post-implantation. (**a**) Representative images of paraffin sections of the indicated BHV tissues after H&E staining. Original magnification ×100. (**b**) Mean ± SEM of scores assigned to describe the amount of cellular immune infiltrate (low = 1, modest = 2, medium = 3, high = 4 and very high = 5) for the studied BHVs (ATS 3f (*n* = 8), Crown (*n* = 8), Pericarbon Freedom/Freedom Solo (Freedom, *n* = 7), Hancock II (Hancock, *n* = 7), and human pericardium (peric., *n* = 7). Statistical differences were found by one-way ANOVA with Tukey’s test between the human pericardium and ATS 3f, Crown, and Freedom as indicated (* *p* ≤ 0.05, ** *p* ≤ 0.005).

Next, a new set of experiments was performed in adult Gal KO mice by grafting subcutaneously a piece per mouse of different commercial BHVs comprising tissues of equine, bovine and porcine origin, as well as glutaraldehyde-fixed human pericardium for comparison (Figure 3). The experiment was ended at the 2-month time point. No major changes were seen in body weight at all study time points (Table 3).

Pathologic studies of the grafts at two months post-implantation were also conducted. As observed in the pilot experiment, the initial macroscopic examination showed that the structure of the BHV tissues was basically preserved in all cases (Figure 1). Signs of immune rejection were not clearly identified macroscopically. However, it was observed that the area of implantation was cloudy in the tissue surrounding the Freedom, the Hancock II, and also the human pericardium implants, whereas it was consistently clean around the Crown grafted pieces and something in between in the ATS 3f implants (Figure 1). Often, the draining lymph nodes became more evident and engrossed in recipients that displayed a reaction to the tissue (Figure 1). The histological analysis revealed that most BHV samples showed a certain degree of cellular immune infiltration that progressed from the edges (Figure 3a). After establishing a 1-to-5 scoring system to better assess and display the results, we found that the strongest cellular immune response was triggered by the glutaraldehyde-fixed human pericardium, followed by the Hancock II (Figure 3a,b). In agreement with the macroscopic findings, the Crown showed an overall lower cellular immune response. Although no statistical differences were reached between the scores of the various commercial BHVs assessed, all but the Hancock II exhibited significantly lower scores than the fixed human pericardium (Figure 3b).

### 3.3. Gal KO Mice Develop an Anti-Gal Antibody Response against Specific BHV Tissues

**Figure 4:**
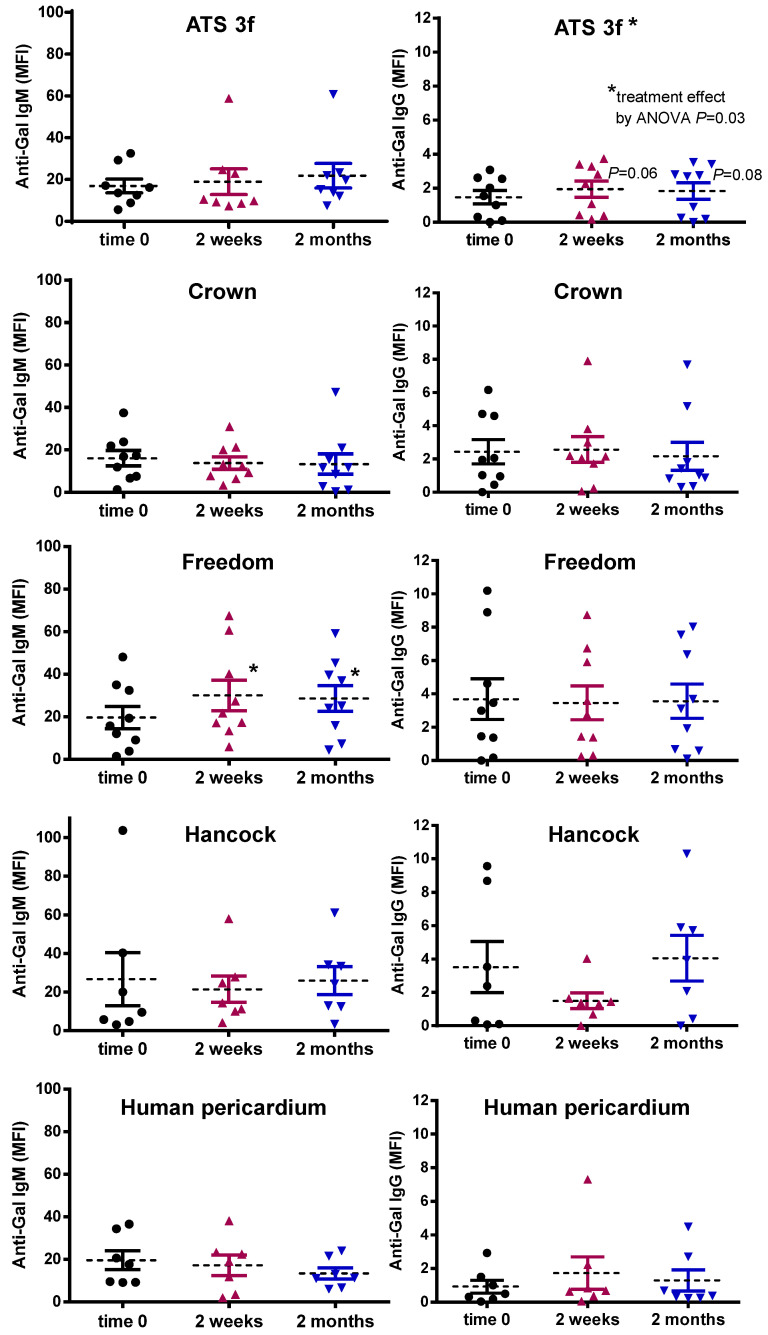
Serum anti-Gal antibody levels in adult Gal KO mice grafted with BHV tissues. Anti-Gal IgM and IgG levels were measured by flow cytometry of PAEC incubated with the various mouse sera alone or with saturating concentrations of GAS914 to set up the background. The average MFI ± SEM of anti-Gal IgM and IgG reactivity after subtracting the background of each determination is shown for each cohort of mice implanted with pieces of ATS 3f (*n* = 9), Crown (*n* = 9), Freedom Solo or Pericarbon Freedom (Freedom, *n* = 9), and *n* = 7 for Hancock II (Hancock) and human pericardium. Statistical differences were found by one-way ANOVA with Dunnett’s test as indicated for increases relative to baseline (time 0) in IgM in mice implanted with Freedom and IgG in mice with ATS 3f (* *p* ≤ 0.05).

The anti-Gal antibody response was also determined in adult Gal KO mice after BHV implantation. Indeed, a very selective antibody response was observed by flow cytometry depending on the implanted tissue. As expected, the Gal-negative human pericardium did not influence the anti-Gal antibody levels (Figure 4). Instead, the ATS 3f tissues led to a mild elevation in reactivity for the IgG at 2-week and 2-month time points relative to a baseline that achieved statistical significance (Figure 4). Notably, grafting of the Freedom tissues in Gal KO mice induced a significant anti-Gal IgM response that, interestingly, was not associated with statistically significant elevations in anti-Gal IgG (Figure 4). By contrast, the anti-Gal antibody profile (IgM and IgG) of mice with Crown tissue after implantation did not significantly differ from baseline and was comparable to that of the human pericardium. Furthermore, no increase in anti-Gal antibodies was found after the implantation of Hancock II tissue. Altogether, these data suggest that the elimination of the Gal antigen in originally Gal-positive tissues could reduce their immunogenicity.

### 3.4. Lack of Correlation between Cellular and Induced Anti-Gal Antibody Responses to BHV Tissues

**Figure 5:**
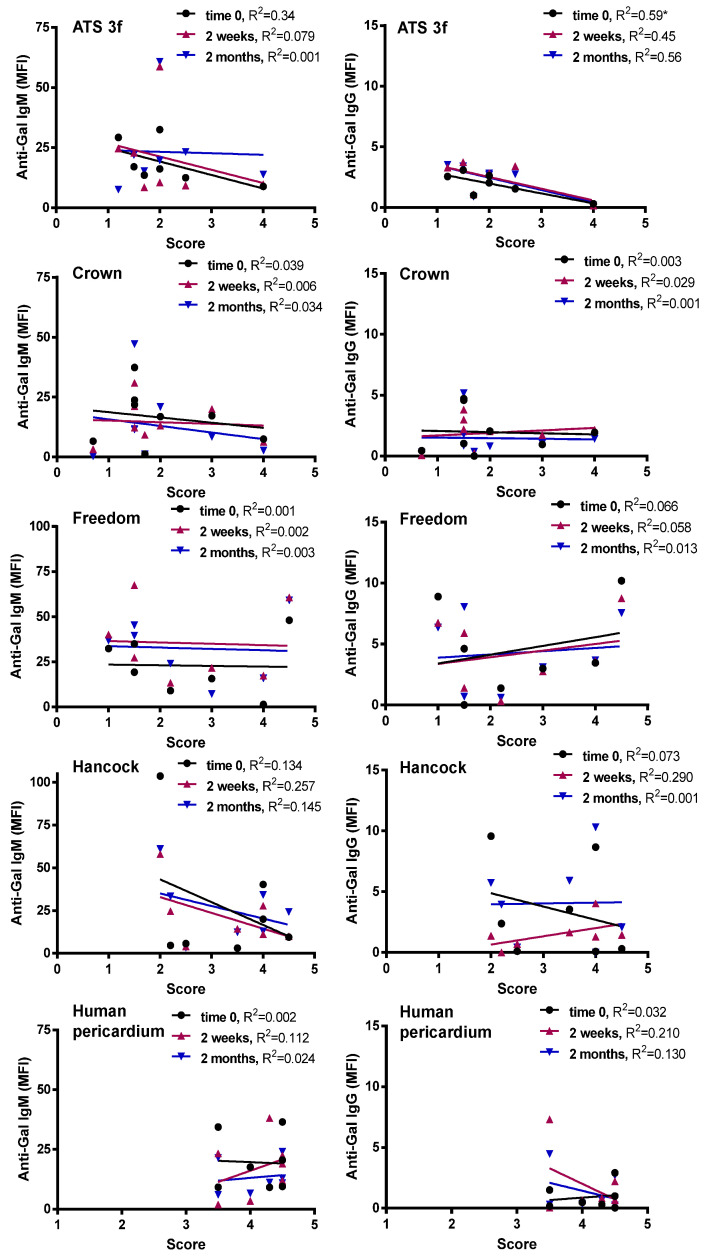
Correlations of anti-Gal antibody reactivities with histological scores in adult Gal KO mice grafted with BHV tissues. The correlation between the histological score and the MFI of anti-Gal IgM and IgG reactivity at the three time points studied was calculated for each cohort of implanted adult mice [ATS 3f (*n* = 8), Crown (*n* = 8), Freedom Solo/Pericarbon Freedom (Freedom, *n* = 7), Hancock II (Hancock, *n* = 7), and human pericardium (*n* = 7)]. The coefficient of determination (R^2^) was obtained by Pearson r (* *p* ≤ 0.05).

Looking at a potential link between the antibody response and the outcome of the implanted BHV tissue, we assessed whether the serum anti-Gal antibody reactivity (determined by flow cytometry) correlated with the histology scores on the cellular immune infiltrate (Figure 5). In general, no correlations were found between the amount of cellular immune infiltrate and the serum anti-Gal antibodies for the multiple BHV tissues at the various time points studied. Exceptionally, an inverse correlation was observed between the anti-Gal IgG (time 0) and the ATS-3f scores (Figure 5).

### 3.5. Gal KO Mice Develop an Anti-Non-Gal IgG Response against Specific BHV Tissues

**Figure 6:**
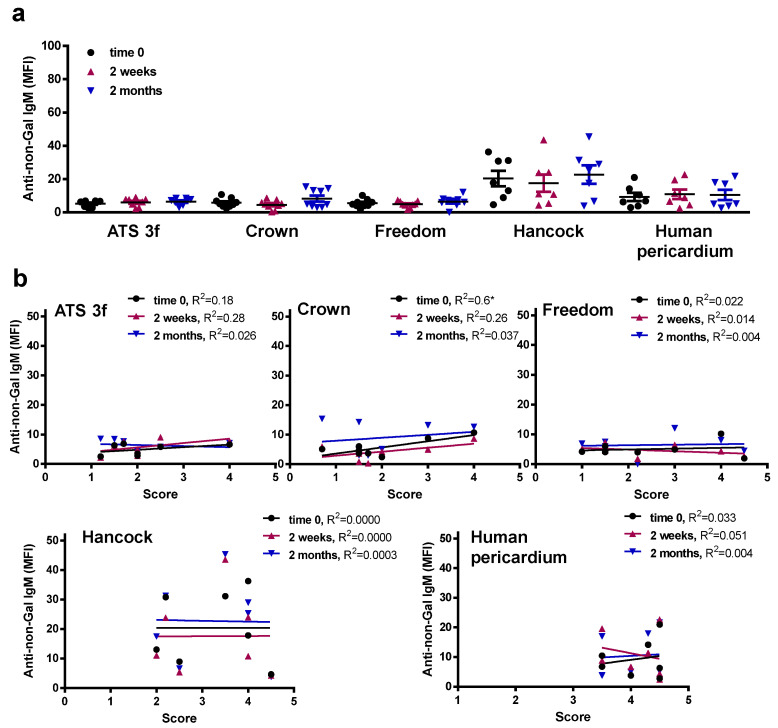
Serum anti-non-Gal IgM levels in adult Gal KO mice grafted with BHV tissues. Anti-non-Gal IgM levels were measured by flow cytometry of PAEC incubated with the various mouse sera with saturating concentrations of GAS914. Reactivity of the anti-mouse IgM antibody was used to set up the background. (**a**) The average MFI ± SEM of anti-non-Gal IgM reactivity after subtracting the background of each determination is shown for each cohort of adult mice implanted with pieces of ATS 3f (*n* = 9), Crown (*n* = 9), Freedom Solo or Pericarbon Freedom (Freedom, *n* = 9), and *n* = 7 for Hancock II (Hancock) and human pericardium. No statistical differences were found after applying the one-way ANOVA with Dunnett’s test. (**b**) The correlation between the histological score and the MFI of anti-non-Gal IgM reactivity at the three time points studied was calculated for each cohort of implanted adult mice [ATS 3f (*n* = 8), Crown (*n* = 8), Freedom Solo/Pericarbon Freedom (Freedom, *n* = 7), Hancock II (*n* = 7), and human pericardium (*n* = 7)]. The coefficient of determination (R^2^) was obtained by Pearson r (* *p* ≤ 0.05).

**Figure 7:**
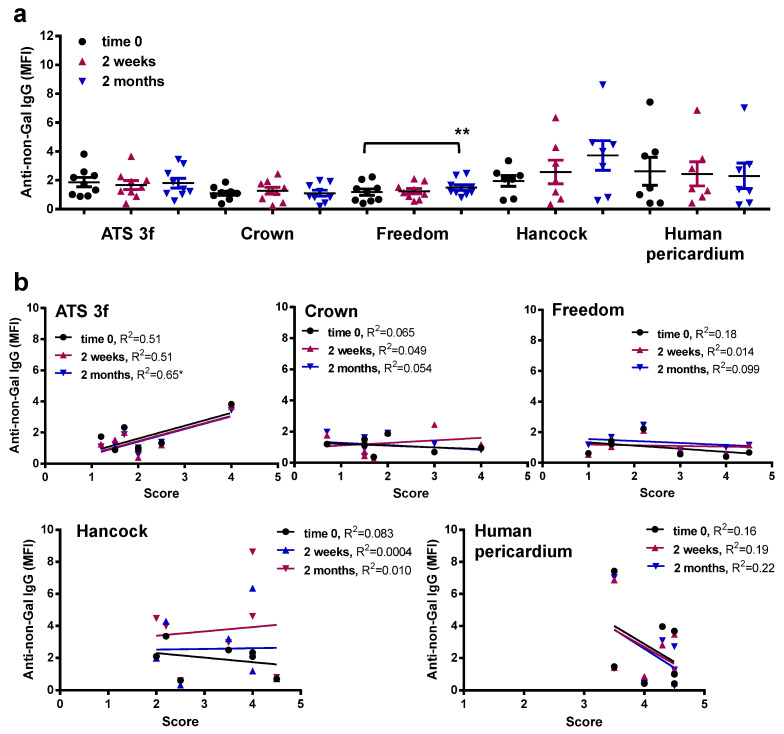
Serum anti-non-Gal IgG levels in adult Gal KO mice grafted with BHV tissues. Anti-non-Gal IgG levels were measured by flow cytometry of PAEC incubated with the various mouse sera with saturating concentrations of GAS914. Reactivity of the anti-mouse IgG antibody was used to set up the background. (**a**) The average MFI ± SEM of anti-non-Gal IgG reactivity after subtracting the background of each determination is shown for each cohort of adult mice implanted with pieces of ATS 3f (*n* = 9), Crown (*n* = 9), Freedom Solo or Pericarbon Freedom (Freedom, *n* = 9), and *n* = 7 for Hancock II (Hancock) and human pericardium. A statistical difference was found only with increased reactivity in mice implanted with Freedom relative to baseline (time 0) by one-way ANOVA with Dunnett’s test (** *p* ≤ 0.005). (**b**) The correlation between the histological score and the MFI of anti-non-Gal IgG reactivity at the three time points studied was calculated for each cohort of implanted adult mice [ATS 3f (*n* = 8), Crown (*n* = 8), Freedom Solo/Pericarbon Freedom (Freedom, *n* = 7), Hancock II (Hancock, *n* = 7), and human pericardium (*n* = 7)]. The coefficient of determination (R^2^) was obtained by Pearson r (* *p* ≤ 0.05).

In the same flow cytometric assays, an anti-non-Gal antibody reactivity was determined for Gal KO mice implanted with BHV tissues using GAS914 pre-treated sera. No significant anti-non-Gal IgM responses were detected for Gal KO mice following this procedure (Figure 6a). Only a mild correlation was found of unclear biological significance between anti-non-Gal IgM reactivities at time 0 and the scores of the retrieved Crown tissue (Figure 6b). However, a significant increase in the reactivity of anti-non-Gal IgG was observed in mice with Freedom tissue at 2 months post-implantation, an elevation that did not correlate with the histological scores (Figure 7a,b). Conversely, the ATS 3f did not elicit a consistent anti-non-Gal IgG response, despite showing a weak correlation with the histological scores (Figure 7a,b). Altogether, the data show that serum anti-non-Gal IgG responses can be detected in this model without proof of their contribution to cellular immunity against BHVs.

## 4. Discussion

In this work, we found that the cellular and antibody responses induced by the implantation of BHV tissues follow separate mechanisms that may differently affect the integrity of implanted BHVs. This finding provides insights into how the immune response may contribute to SVD and points to the need for multiple strategies of intervention to address SVD. The cell- and antibody-mediated pathways acted as independent factors in the adult Gal KO mouse model. Notably, the anti-Gal and anti-non-Gal antibody responses in BHV-implanted patients have been recently related to BHV calcification and, thus, to SVD [14,21,22,23,24]. Therefore, Gal KO mice could be a valuable preclinical tool to gain further insight into the immunological mechanisms of SVD and facilitate the development of better BHVs. The fact that some commercial BHVs trigger a stronger cellular (Hancock II) or antibody (Freedom/Solo) response in this mouse model might help to predict the clinical outcomes. Furthermore, our findings emphasize the need to specifically study the immune response against each BHV tissue type, whether it is under development or in clinical use.

A cellular immune response was observed by histopathological analysis for all BHV tissues at 2 months post-implantation, a time period that was found to be appropriate to assess the cellular immune infiltrate in the pilot experiments. The macroscopic examination did not provide much information, but the histological analysis revealed the presence of macrophages, lymphocytes, and plasma cells surrounding the grafted tissue at this time point. Notably, macrophages and lymphocytes have also been observed in BHV retrieved from patients [34,35]. The mechanisms responsible for the immune infiltrates in SVD are not fully understood, but various pathways have been proposed, including the release of proteolytic enzymes and reactive oxygen species [12,13,14,15,35,36]. In the present study, the samples that displayed a prominent and penetrating cellular immune infiltrate were associated with more severe structural alterations. Studies in Gal KO mice with glutaraldehyde-fixed bovine pericardium implanted subcutaneously also described the presence of a mononuclear-cellular immune infiltrate containing macrophages and T cells [37]. Interestingly, this group reported that the cellular immune response is amplified in the grafts of Gal KO mice as compared with wild-type mice [37,38], a finding that was also observed in our Gal KO model when grafted with Gal-positive living (cartilage) xenografts [30].

The semi-quantitative differences in the cellular immune infiltrates between the various tissues studied were relevant findings of the present work. The fixed human pericardium, followed by the Hancock II (porcine), triggered the highest cellular immunity. The other tissues assessed, including the ATS 3f, the bovine Freedom/Solo tissue, and the Crown (bovine pericardium with a phospholipid reduction treatment), induced a lower cellular response. Although neither commercial BHVs nor human pericardium has been previously studied in this Gal KO model, other studies evaluated the cellular immune responses to various glutaraldehyde-processed pericardial tissues at 2 and 3 months post-implantation [37,38,39]. In those studies, decellularization or anti-calcification treatments decreased the calcium content in the tissues but not the amount of cellular immune infiltrate [37,38,39]. In our study, the strong cellular immune response against human pericardium was observed in the absence of an induced anti-carbohydrate antibody response as determined by our assay with PAEC. This is consistent with the direct activation of macrophages, lymphocytes, and platelets by glutaraldehyde tissue fixation [35].

Major differences were also found in the antibody responses to the various tissues studied. Evidence for the role of antibodies in SVD has been provided experimentally by demonstrating the contribution of anti-Gal and anti-Neu5Gc antibodies to the calcification process of BHV tissues in various animal models [14,40]. In our study, the anti-Gal antibodies were increased only after the implantation of ATS 3f and Freedom/Solo tissue. Anti-non-Gal IgG was also elevated by Freedom/Solo implants, which readily showed the presence of plasma cells in histological analyses. No elicited xenoantibody response was determined in recipients of Crown or Hancock II tissue. Previous comparative analyses of specific antibody responses to BHV are limited to a single and small clinical study that found no difference in anti-Gal antibodies between patients with bovine (Carpentier-Edwards Perimount) and porcine (Hancock II and Epic Supra combined) BHVs [24]. Furthermore, the antibody response was not differentially affected by the BHV species of origin in the Translink clinical study that included patients with a bovine, porcine, or equine BHV [14]. Other studies in the Gal KO model do not compare commercial BHVs, and their anti-Gal antibody determinations by ELISA are difficult to interpret [37,38,39]. Thus, our experimental model may provide information of interest to the clinician regarding the different immunogenicity of commercial BHVs. The anti-Gal antibody response we observed for the ATS 3f tissues probably results from its high Gal-antigen content, as determined by IB4 reactivity [18]. However, it is yet unclear why the Freedom/Solo tissue triggers such a strong antibody response in this model. This tissue is fixed with glutaraldehyde and processed with homocysteic acid as an anti-calcification treatment. It can be hypothesized that this procedure allows high exposure of carbohydrate antigens that are directly recognized by B cells leading to T-independent activation, plasma cell generation, and IgM secretion [30]. By contrast, based on our own observations, the treatment of Crown with octanediol might be efficacious in preventing a xenoantibody response.

Positive correlations between serum antibody reactivity and histological scores were rarely observed in this study. Exceptionally, a correlation was found between anti-non-Gal IgG reactivity and the histological scores of ATS 3f at two months post-implantation despite these antibodies not reaching a statistically significant elevation at this time point. Nevertheless, the higher amount of cellular immune infiltrate did not generally coincide with increases in specific serum antibodies. In the mouse model used, there were situations that elicited a specific antibody response, others with a predominant cellular immune response, and even a mixed profile was observed. Intriguingly, a statistically significant negative correlation was found between pre-implantation anti-Gal IgG and the histological score for ATS 3f grafts but not for other BHV tissues. Elucidating the mechanism behind this is beyond the scope of this work, but we can hypothesize that it may be caused by the variability of Gal KO mice in Th1/Th2 balances that influence the anti-Gal IgG natural antibody titers and the cellular immune response to the ATS 3f tissue in opposite ways. In summary, our data support the concept of at least two separate immune pathways (antibody- and cell-mediated) participating in BHV-tissue deterioration.

Based on our data, it should be considered that some BHV tissues may trigger a response with Th1 predominance (cellular) while others may induce a more Th2-driven (humoral) reaction. Furthermore, innate immunity and inflammation, as represented by the well-characterized biomarker C reactive protein, may contribute to these processes and be of interest for inclusion in future studies [41]. Indeed, it will be interesting to know how all these mechanisms influence the clinical outcomes and, at the same time, highlights the preclinical value of our Gal KO mouse model to assess the immune response against the BHV tissues. The lack of anti-calcification treatment and the human-to-mouse xenogeneic combination probably caused the strong cellular response to the human pericardium in this model. Notably, our findings of a predominant cell-mediated deterioration of Hancock II and a mixed pattern in the pericardial tissues are in keeping with previous descriptions of the type of BHV failure in the clinical setting [42,43]. Particularly, Grunkemeier et al. found a higher incidence of tears for a BHV made with pig valves compared to a bovine pericardial BHV from the same manufacturer [42], and Kwak et al. described more shrinkage or tearing in explanted Hancock II and higher calcification and fibrosis for the bovine pericardial BHV [43]. We can speculate that this may be caused by the differential immune response. Among the bovine pericardial BHVs, the worse clinical outcomes have been described for the Mitroflow without anti-calcification treatment [10], but poor long-term function has also been reported for the Freedom Solo [44]. In fact, variable effects have been described for this BHV, with some patients suffering BHV tears whilst others present severe BHV calcification and stenosis [44]. The high and complex immunogenicity of this tissue in the model used would be consistent with these clinical findings. Regarding the pericardial equine BHVs, there is insufficient data reported for the long-term clinical assessment of ATS 3f and Enable 3f (same tissue), but it would be of interest to contrast it with our data in future reports. Likewise, the clinical experience with Crown is short [45], and it will be restricted by the recent termination of its distribution in Europe.

## 5. Conclusions

We found high variability in the induction of cellular and antibody responses by the different commercial BHV tissues implanted subcutaneously in adult Gal KO mice. Penetrating cellular immunity was associated with stronger tissue deterioration. However, no major correlations between the amount of cellular immune infiltrate in the graft and the elicited serum antibody levels were observed. Summarily, it may be concluded that the cellular and antibody responses are independent factors in BHV deterioration in the Gal KO mouse model. This model may provide valuable information for predicting the immunogenicity of commercial and newly developed BHVs.

## Figures and Tables

**Table 1:** BHV tissue types, and their processing method, were grafted subcutaneously in adult Gal KO mice to assess the immune response ^1^.

BHV Name	Tissue Source	BHV Treatment	*n*
ATS 3f	Equine pericardium	Glutaraldehyde	10
CROWN	Bovine pericardium	Glutaraldehyde + Octanediol	9
FREEDOM	Bovine pericardium	Glutaraldehyde + Homocysteic acid	10
HANCOCK	Porcine aortic valves	Glutaraldehyde + Sodium dodecyl sulfate	7
huPERIC	Human pericardium	Glutaraldehyde	7

^1^ Adult Gal KO mice were implanted subcutaneously with a piece of tissue of ATS 3f, Crown, Freedom Solo or Pericarbon Freedom (Freedom), Hancock II, or human pericardium (huPERIC,). The implants were free of any additional material from the BHVs (e.g., sutures, scaffolding material). *n*: number of total experimental mice used per cohort.

**Table 2:** Body weight (g, mean ± SEM) of adult Gal KO mice grafted subcutaneously with BHV tissues for 4 months ^1^.

BHV	Baseline	3 Days	3 Weeks	2 Months	4 Months
ATS 3f	34.8 ± 0.9	33.2 ± 1 *	32.9 ± 1	34 ± 0.6	32.8 ± 0.6
FREEDOM	34.7 ± 1.1	32.7 ± 1.3 *	34 ± 1.2	33.3 ± 1.4	33.3 ± 1.1

^1^ Adult Gal KO mice were grafted with either ATS 3f (*n* = 6) or Freedom Solo (Freedom, *n* = 6), and the body weight (g) was recorded at the indicated time points relative to implantation. Statistical significance by one-way ANOVA with Dunnett’s test relative to baseline is shown (* *p* ≤ 0.05).

**Table 3:** Body weight (g, mean ± SEM) of adult Gal KO mice grafted subcutaneously with BHV tissues for 2 months ^1^.

BHV	Baseline	2 Weeks	2 Months
ATS 3f	35.6 ± 0.5	34.6 ± 0.8	34.6 ± 1.1
CROWN	35.7 ± 0.8	34.7 ± 0.9	33.7 ± 1.3 *
FREEDOM	32.8 ± 1.5	32.7 ± 1.1	33.4 ± 1.2
HANCOCK	36.3 ± 0.8	35.3 ± 0.9	34.6 ± 0.3
huPERIC	36.4 ± 1.3	35.7 ± 1.4	35.8 ± 1.2

^1^ Adult Gal KO mice received either ATS 3f (*n* = 9), Crown (*n* = 9), Freedom Solo (Freedom, *n* = 10) Hancock II (*n* = 7), or human pericardium (huPERIC, *n* = 7) and the body weight (g) was recorded at the indicated time points relative to implantation. Statistical significance by one-way ANOVA with Dunnett’s test relative to baseline is shown (* *p* ≤ 0.05).

## Data Availability

Data are contained within the article or Supplementary Material.

## Supplementary Materials

The following supporting information can be downloaded at: https://www.mdpi.com/article/10.3390/bioengineering10070833/s1, Figure S1: Determination of serum anti-Gal antibody reactivity in Gal KO mice grafted with a piece of BHV. Mouse sera isolated at various time points were incubated at 0.5% in plate wells coated Gal-HSA and IgM (A) and IgG (B) reactivity was measured by absorbance at 492 nm. Mice 1 to 3 (orange range) were grafted with ATS 3f and mice 4–6 (green range) received Pericarbon Freedom. Baseline reactivity (before implantation) is shown at time 0.; Figure S2: Determination of serum anti-PAEC antibody reactivity in Gal KO mice grafted with a piece of BHV. Mouse sera isolated at various time points were incubated at 1% (A) or 0.5% (B) with PAEC alone or with saturating concentrations of GAS914 and IgM (A) and IgG (B) reactivities were measured by FACs. The MFI after subtracting the background of secondary antibody is shown for each mouse. Mice 1 to 3 were grafted with ATS 3f (orange range) and mice 4–6 received Pericarbon Freedom (green range); Table S1: Anti-PAEC antibody reactivity by sera from adult Gal KO mice with BHV subcutaneous implants for 4 months.
