# Supplementary material for: Differential Immune Response to Bioprosthetic Heart Valve Tissues in the α1,3Galactosyltransferase-Knockout Mouse Model
Source: Bioengineering (Basel). 2023 Jul 13;10(7):833. doi: 10.3390/bioengineering10070833 (PMC10376745; doi:10.3390/bioengineering10070833)
Supplement: Supplementary file 1 [file bioengineering-10-00833-s001.zip › bioengineering-2450122-supplementary.pdf]

bioengineering

MDPI

*Supplementary Materials*

# Differential Immune Response to Bioprosthetic Heart Valve Tissues in the $\alpha$1,3Galactosyltransferase-Knockout Mouse Model

**Kelly Casós [1], Roger Llatjós [2], Arnau Blasco-Lucas [3], Sebastián G. Kuguel [1], Fabrizio Sbraga [3], Cesare Galli [4], Vered Padler-Karavani [5], Thierry Le Tourneau [6], Marta Vadori [7], Andrea Perota [4], Jean-Christian Roussel [6], Tomaso Bottio [8], Emanuele Cozzi [7], Jean-Paul Soulillou [9], Manuel Galiñanes [10], Rafael Máñez [1,11] and Cristina Costa [1,*]**

1 Infectious Diseases and Transplantation Division, Institut d'Investigació Biomèdica de Bellvitge [IDIBELL], L'Hospitalet de Llobregat, 08908 Barcelona, Spain
2 Pathology Department, Bellvitge University Hospital, L'Hospitalet de Llobregat, 08907 Barcelona, Spain
3 Cardiac Surgery Department, Bellvitge University Hospital, L'Hospitalet de Llobregat, 08907 Barcelona, Spain
4 Avantea Srl, 26100 Cremona, Italy
5 Department of Cell Research and Immunology, The Shmunis School of Biomedicine and Cancer Research, The George S. Wise Faculty of Life Sciences, Tel Aviv University, Tel Aviv 6997801, Israel
6 Institut du Thorax, INSERM UMR1087, Nantes University Hospital, 44093 Nantes, France
7 Transplantation Immunology Unit, Padua University Hospital, 35128 Padova, Italy
8 Department of Cardiac, Thoracic, Vascular Sciences and Public Health, University of Padua Medical School, 35121 Padova, Italy
9 Institut de Transplantation-Urologie-Néphrologie, INSERM Unité Mixte de Recherche 1064, Nantes University Hospital, 44093 Nantes, France
10 Department of Cardiac Surgery and Reparative Therapy of the Heart, Vall d'Hebron Research Institute [VHIR], University Hospital Vall Hebron, Universitat Autònoma de Barcelona, 08035 Barcelona, Spain
11 Intensive Care Department, Bellvitge University Hospital, L'Hospitalet de Llobregat, 08907 Barcelona, Spain
* Correspondence: ccosta_valles@yahoo.com

A

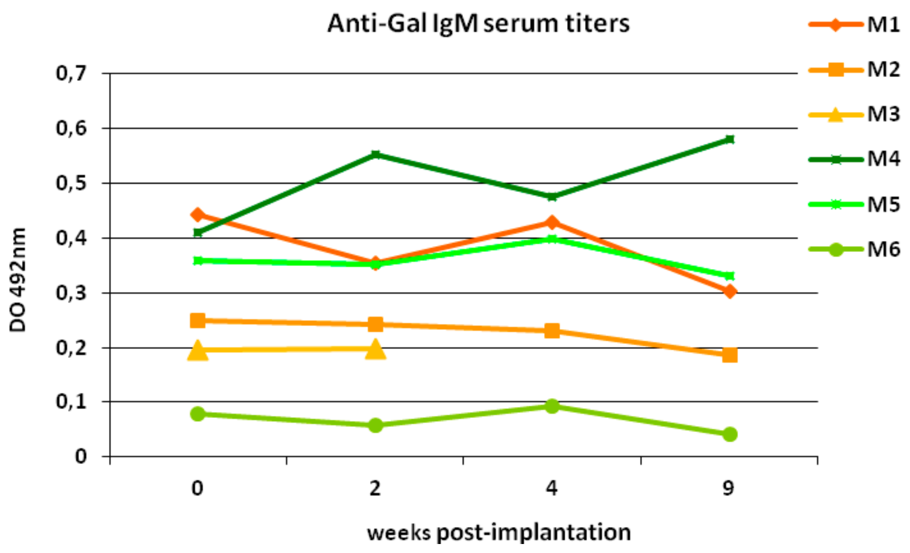


B

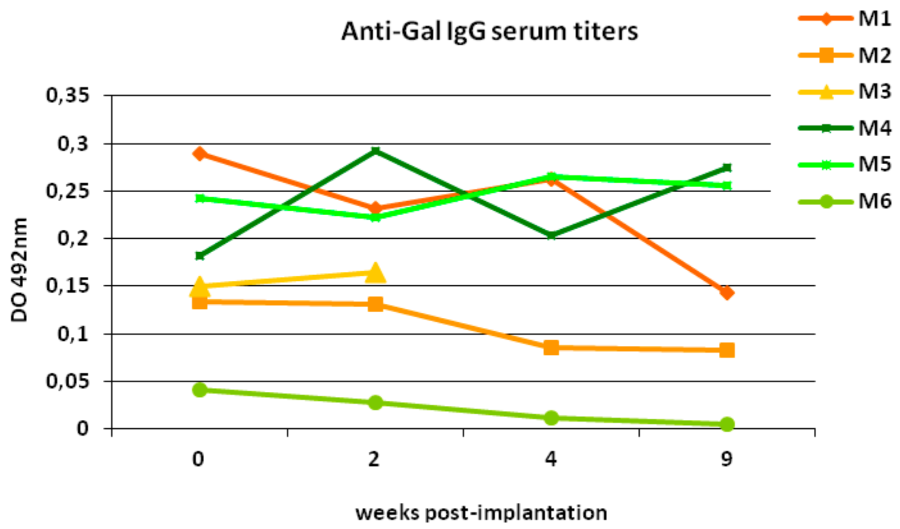


**Figure S1.** Determination of serum anti-Gal antibody reactivity in Gal KO mice grafted with a piece of BHV. Mouse sera isolated at various time points were incubated at 0.5% in plate wells coated Gal-HSA and IgM (**A**) and IgG (**B**) reactivity was measured by absorbance at 492 nm. Mice 1 to 3 (orange range) were grafted with ATS 3f and mice 4-6 (green range) received Pericarbon Freedom. Baseline reactivity (before implantation) is shown at time 0.

A

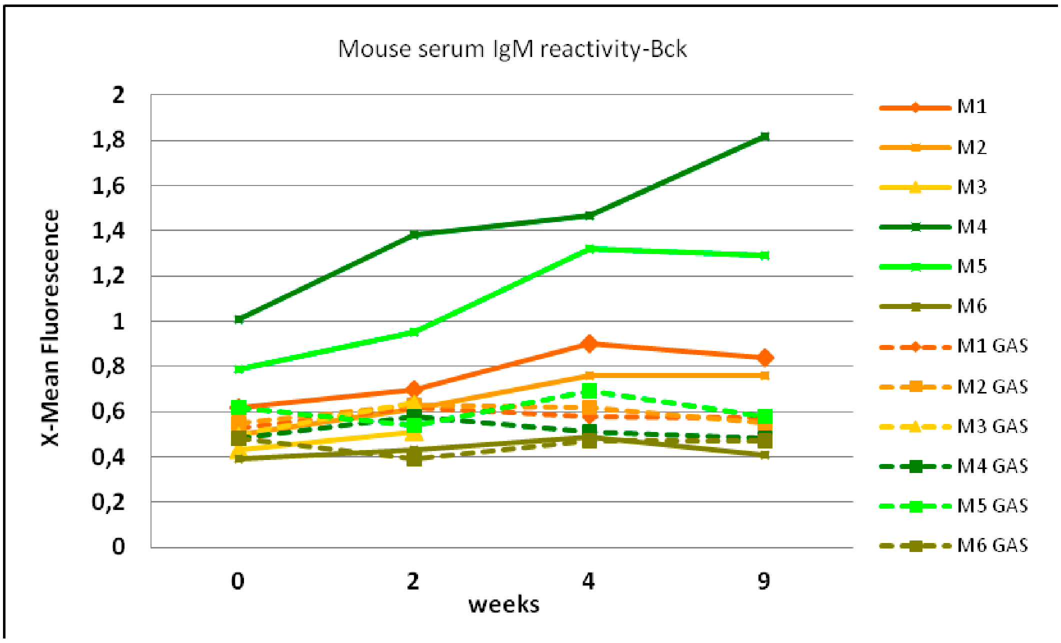


B

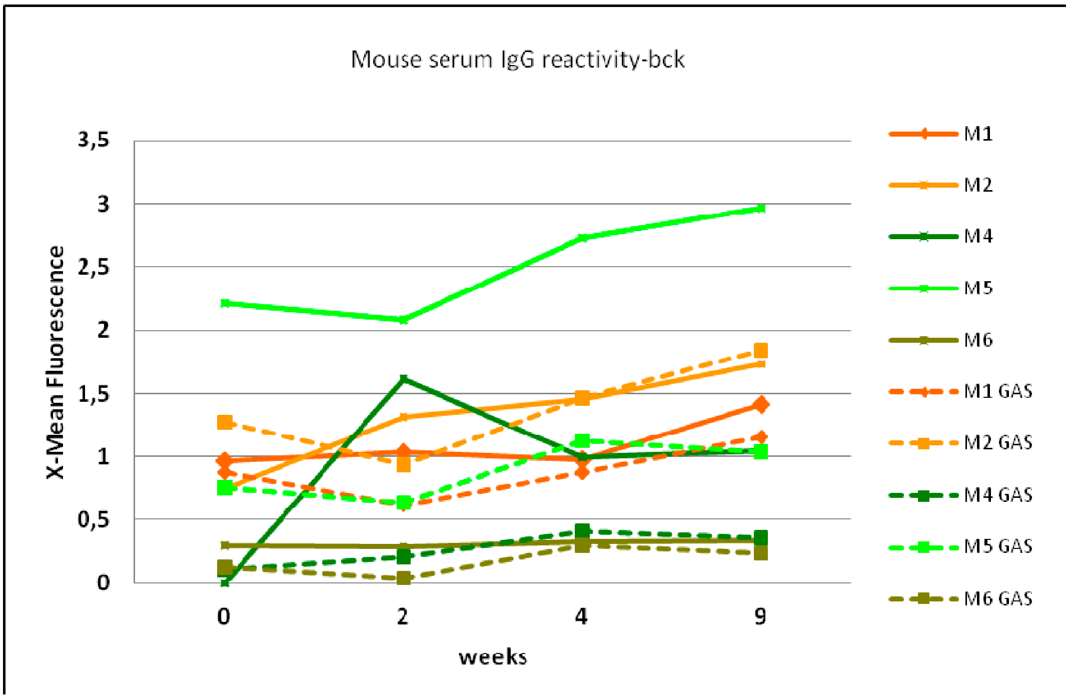


**Figure S2.** Determination of serum anti-PAEC antibody reactivity in Gal KO mice grafted with a piece of BHV. Mouse sera isolated at various time points were incubated at 1% (**A**) or 0.5% (**B**) with PAEC alone or with saturating concentrations of GAS914 and IgM (A) and IgG (B) reactivities were measured by FACs. The MFI after subtracting the background of secondary antibody is shown for each mouse. Mice 1 to 3 were grafted with ATS 3f (orange range) and mice 4-6 received Pericarbon Freedom (green range).

**Table S1.** Anti-PAEC antibody reactivity by sera from adult Gal KO mice with BHV subcutaneous implants for 4 months.

| | time 0 | 3 weeks | 2 months | 4 months |
|---|---|---|---|---|
| **ATS 3f IgM** | 17.5 ± 3.2 | 15.9 ± 2.9 | 13.5 ± 3.2 | 15.5 ± 3.1 |
| **ATS 3f IgG** | nd | nd | nd | nd |
| **FREEDOM IgM** | 19.7 ± 7.2 | 22.5 ± 6.3 | 23.6 ± 7.5 | 20.6 ± 6.2 |
| **FREEDOM IgG** | 6.7 ± 3 | 7.2 ± 3.2 | 10.3 ± 3.9 | 8.5 ± 2.1 |

Data are presented as Mean ± SEM of mean fluorescence intensity (n = 6 mice/cohort) without further normalization. Individual mouse sera isolated at the indicated time points post-implantation were incubated at 1% and 0.5% with PAEC and IgM and IgG reactivity respectively determined by FACs. Abbreviations: nd, not determined; FREEDOM, Pericarbon Freedom/Freedom Solo.
